# A discrete mathematical model for the aggregation of *β-Amyloid*

**DOI:** 10.1371/journal.pone.0196402

**Published:** 2018-05-23

**Authors:** Maher A. Dayeh, George Livadiotis, Saber Elaydi

**Affiliations:** 1 Department of Space Research, Southwest Research Institute, San Antonio, Texas, United States of America; 2 Department of Mathematics, Trinity University, San Antonio, Texas, United States of America; University of Florida, UNITED STATES

## Abstract

Dementia associated with the Alzheimer's disease is thought to be correlated with the conversion of the β − Amyloid (Aβ) peptides from soluble monomers to aggregated oligomers and insoluble fibrils. We present a discrete-time mathematical model for the aggregation of Aβ monomers into oligomers using concepts from chemical kinetics and population dynamics. Conditions for the stability and instability of the equilibria of the model are established. A formula for the number of monomers that is required for producing oligomers is also given. This may provide compound designers a mechanism to inhibit the Aβ aggregation.

## Introduction

Alzheimer's disease (AD) is the most cause of dementia and the most prevalent neurodegenerative disorder [1, 2, 3]. It is a progressive degenerative disorder that is age-related and is characterized by the loss of synapses and neurons from the brain and by the presence of extracellular protein-containing deposits (senile plaques) and intracellular neurofibrillary tangles [4, 2]. Β − Amyloid is the principal protein component of the extracellular plaques. Aβ is a 39- to 43- residue proteolytic product of a parental amyloid precursor protein (APP) that localizes to the plasma membrane, trans-Golgi network, endoplasmic reticulum (ER) and endosomal, lysosomal and mitochondrial membranes [5, 6]. Aβ contains sequences from extracellular and transmembrane regions of the parent protein [7, 8]. The spontaneous conversion of Aβ monomers into febrillar aggregates is found to be associated with the development of AD [9]. In fact, the neurodegenerative effects of AD are hypothesized to arise from Aβ. This is commonly known as the amyloid hypothesis, and is the dominant model of AD pathogenesis [10, 11, 5, 12, 13, 14, 15]. Albeit, amounting support from biochemical, genetic, and transgenic animal studies that supports the amyloid hypothesis, [16, 17, 18, 19, 20], debate over the amyloid hypothesis remains controversial.

In general, protein aggregation from soluble to non-soluble structures has been linked to be a causative factor of several diseases, including AD, Parkinson's disease, Huntington's disease, Prion disease, among others [21, 15]. For the case of Aβ linkage to AD, it has been found that Aβ becomes indeed toxic once aggregated [22, 23, 24, 25, 26]. Furthermore, numerous studies show a strong correlation between soluble Aβ oligomer levels and the extent of synaptic loss [27, 28, 29, 30, 31, 32], further suggesting that the soluble oligomers are the causative agents of AD [33, 34, 35, 36]. This in turn has motivated several studies aimed at exploiting Aβ aggregation mechanisms and kinetics of Aβ conversion. Especially that synthetic Aβ was found to spontaneously aggregates into sheet-rich fibrils, resembling those in plaques [5].

Naiki and Nakakuki [37] proposed a simple mathematical model in which fibril elongation is postulated to occur by reversible addition of monomers to preexisting fibrils. The model, however, does not explain the generation of new fibrils neither it simulates fibril length. Lomakin [38] proposed a detailed kinetic model in which they postulated that rapid reversible equilibrium between monomers and micelles occurs, followed by spontaneous generation of nuclei from micelles, in an irreversible process. Fibrils then grew by adding monomers to the fibril tip or the nucleous. The model accounts for the co-existence of monomers and fibrils and is capable of predicting fibril evolution (mass and length) in time. However, the experiments leading to the model development were performed in non-physiological conditions (pH 1). Pallitto [3] developed a kinetic model that qualitatively de-scribed Aβ self-association kinetics from the unfolded state. The model incorporated information about mass distribution and length changes of Aβ and accounted for the co-existence of monomer, dimer, and aggregated species. The model provided mechanisms for both generation and elongation of fibrils, and was able to capture all the essential features of the experimental data.

The structure of the Aβ monomer is difficult to characterize due to its tendency to aggregate. Experiments focusing on the understanding of physical structure of Aβ showed that they are not necessarily homogenous in shape. Filament (3–4 nm in diameter) and fibril (8–10 nm in diameter) structures have been observed in several electron microscopy and atomic force microscopy experiments [39, 40, 26]. Furthermore, Malinchik et al. [41] suggested that fibers are made of three to five laterally associated filaments. Fraser [42] reported on observing amyloid fibers made of five to six globular units, each with 2.5–3 nm in diameter. Finally, Reixach et al. [43] showed that oligomers are formed from the aggregation of at most six monomers.

Mathematical models of the Aβ kinetics provide a clearer mechanistic understanding of the amyloid fibril growth, improve our ability to design compounds that alter and modulate fibril formation, and provide therapeutic potential venues for the AD.

This paper develops a discrete mathematical model for the aggregation of monomers to oligomers and discusses a mechanism to reduce the production of oligomers. The model is based on the assumption that soluble Aβ oligomers are the causative agents of AD. First, we provide a brief review of chemical kinetics and then proceed to develop the discrete model. A stability analysis follows which determines the aggregation condition. Finally, A formula for the number of monomers that is required for producing oligomers is provided. In [44], Puri and Li developed a continuous-time (differential equation) model focusing on the network cross talk among microglia, neuron, and astroglia, and the corresponding pathological consequence. However, in this work, we take a different approach (discrete) in modeling the aggregation of β − Amyloid into diamers, triamers, etc., and finally into oligomers. The novelty of our approach is the utilization of ideas from chemical kinetics [45] and population dynamics [46], to develop a discrete-time model describing this process.

## A brief review of chemical kinetics

In chemical kinetics [45], if A is the reactant and B is the product, so that A→B, then the average rate of the reaction describes the change in the concentration of either A or B, and is given by,

$$\frac{\Delta A}{\Delta t}=\frac{change\;in\;number\;of\;molectues\;in\;A}{change\;in\;time}$$ (1)

and

$$\frac{\Delta B}{\Delta t}=\frac{change\;in\;number\;of\;molectues\;in\;B}{change\;in\;time}$$ (2)

moreover,

$$\frac{\Delta B}{\Delta t}=-\frac{\Delta A}{\Delta t}$$ (3)

Letting Δ*t* = 1, we obtain from (3),

B(t+1)−B(t)=−(A(t+1)−A(t)) (4)

In general, for the reaction,

aA+bB→cC+dD (5)

where a, b, c, d are the number of molecules of A, B, C, D respectively, we have,

$$average\;rate\;of\;reaction=-\frac{1}{a}\Delta A=-\frac{1}{b}\Delta B=-\frac{1}{c}\Delta C=-\frac{1}{d}\Delta D$$ (6)

The reaction rate law expression relates the rate of an elementary reaction to the concentration of each reactant, that is

$$average\;rate\;of\;reaction=K.A^{a}.B^{b}$$ (7)

where K is the reaction constant, to be determined experimentally.

## The construction of the model: Making oligomers

**Fig 1:**
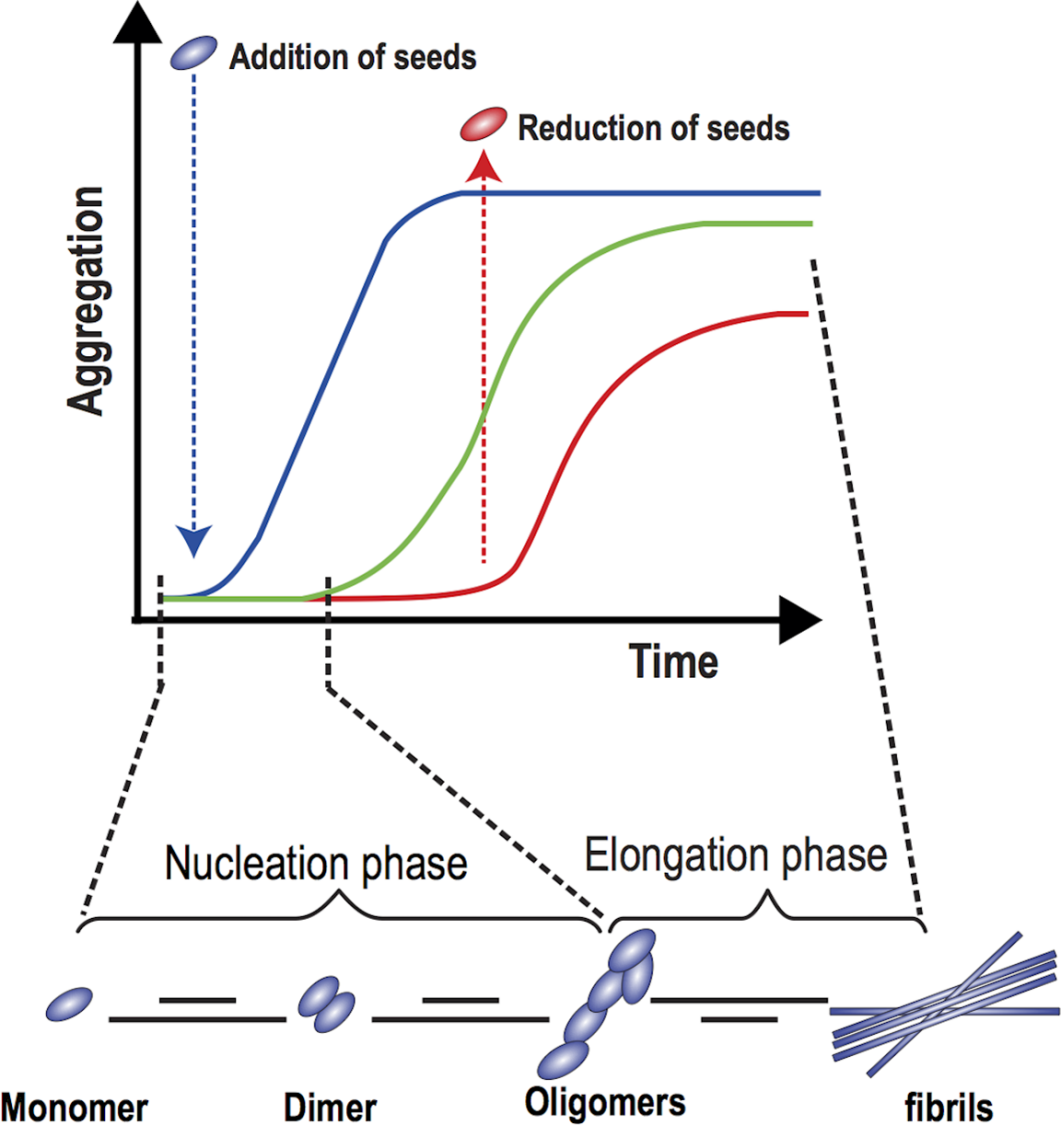
The aggregation of β − Amyloid: From monomers to oligomers. See text for details. Adapted from [48].

In its simplest forms, Amyloid formation can be described by protein aggregation, involving the misfolding of Aβ into soluble and insoluble assemblies [47, 48]. Kinetic studies have suggested that the misfolding of monomeric Aβ has been shown to precede the formation of oligomers, which then serve as seeds for accelerated fibril growth, [49], as illustrated in Fig 1.

The two phases of Amyloid formation are shown: (i) nucleation phase, in which monomers undergo misfolding and associate to form oligomeric nuclei, and (ii) elongation phase, in which the oligomeric nuclei rapidly grow by further addition of monomers, forming larger fibrils. As explained in [48], the nucleation phase occurs gradually and at a slower rate than the elongation phase which proceeds faster being more favorable. A sigmoidal curve can thus describe the process. Addition of more monomers (seeds) speeds up the process and induces faster aggregate formation (blue curve). In contrast, the lack of monomers introduces lag time and slows down the aggregation process.

We now proceed to the modeling part. We assume that (i) monomers of Aβ aggregate to form diamers (2 monomers), triamers (3 monomers), …, etc. (ii) only monomers aggregate with diamers, triamers, …. (iii) this process of aggregation is irreversible., and finally, (iv) oligomers are formed from the aggregation of six monomers.

Let *M*_1_, *M*_2_, *M*_3_,…,*M*_*n*−1_, denote the number of monomers, diamers, triamers,:…, respectively. It is assumed that n monomers aggregate to make an oligomer as shown in Fig 1. A mathematical scenario is illustrated in Table 1.

**Table 1:** Kinetics of monomers aggregation as a function of time.

Reaction	Reaction rate	Change in Δ*M*_1_
*M*_1_ + *M*_1_ → *M*_2_	$-\frac{1}{2}\Delta M_{1}=\Delta M_{2}$	Δ*M*_1_(*t*) = −2*K*_1_*M*_1_(*t*)*M*_1_(*t* + 1)
*M*_1_ + *M*_2_ → *M*_3_	−Δ*M*_1_ = −Δ*M*_2_ = −Δ*M*_3_	Δ*M*_1_(*t*) = −*K*_2_*M*_2_(*t*)*M*_1_(*t* + 1)
*M*_1_ + *M*_3_ → *M*_4_	−Δ*M*_1_ = −Δ*M*_3_ = −Δ*M*_4_	Δ*M*_1_(*t*) = −*K*_3_*M*_3_(*t*)*M*_1_(*t* + 1)
⋮	⋮	⋮
*M*_1_ + *M*_*n*−2_ → *M*_*n*−1_	−Δ*M*_1_ = −Δ*M*_*n*−2_ = −Δ*M*_*n*−1_	Δ*M*_1_(*t*) = −*K*_*n*−2_*M*_*n*−2_(*t*)*M*_1_(*t* + 1)

It is important to note that the change Δ*M*_1_ for each reaction are different. In Table 1 column 3, we determine the change in this concentration for each reaction. Adding Δ*M*_1_ for all reactions, we get, the overall change for *M*_1_:

$$\Delta M_{1}(t)=-{2K}_{1}M_{1}(t)M_{1}(t+1)-K_{2}M_{2}(t)M_{1}(t+1)-K_{3}M_{3}(t)M_{1}(t+1)-\cdots$$

Which can be written as,

$$\Delta M_{1}(t)=-{2K}_{1}M_{1}(t)M_{1}(t+1)-M_{1}(t+1){\sum_{i=2}^{n-1}K_{i}M_{i}(t)}_{\;}$$ (8)

Similarly, for i >1 at the ith reaction, we have,

$$M_{1}+M_{i}\to M_{i+1},\;i>1$$

therefore, the overall change for *M*_i_, i>1:

$$\Delta M_{i+1}(t)=K_{i}M_{i}(t)M_{1}(t)-{K_{i+1}M_{1}(t)M}_{i+1}(t+1)$$ (9)

which can be expanded as,

$$\Delta M_{2}(t)={K_{1}M}_{1}^{2}(t)-K_{2}M_{1}(t)M_{2}(t+1)$$

$$\Delta M_{3}(t)=K_{2}M_{1}(t)M_{2}(t)-K_{3}M_{1}(t)M_{3}(t+1)$$

⋮

$$\Delta M_{n-1}(t)=K_{n-2}M_{1}(t)M_{n-2}(t)-K_{n-1}M_{1}(t)M_{n-1}(t+1)$$

Since monomers are produced by the body, we assume a source function that this is represented by *f*(*M*_1_). Hence the discrete model is finally given by

$$\Delta M_{1}(t)={f(M}_{1}(t))-\;2K_{1}M_{1}(t)M_{1}(t+1)-M_{1}(t+1){\sum_{t=2}^{n-1}K_{i}M_{i}(t)}_{\;}$$

$$\Delta M_{2}(t)={K_{1}M}_{1}^{2}(t)-K_{2}M_{1}(t)M_{2}(t+1)$$

$$\Delta M_{3}(t)=K_{2}M_{1}(t)M_{2}(t)-K_{3}M_{1}(t)M_{3}(t+1)$$

⋮

$$\Delta M_{n-1}(t)=K_{n-2}M_{1}(t)M_{n-2}(t)-K_{n-1}M_{1}(t)M_{n-1}(t+1)$$

**Fig 2:**
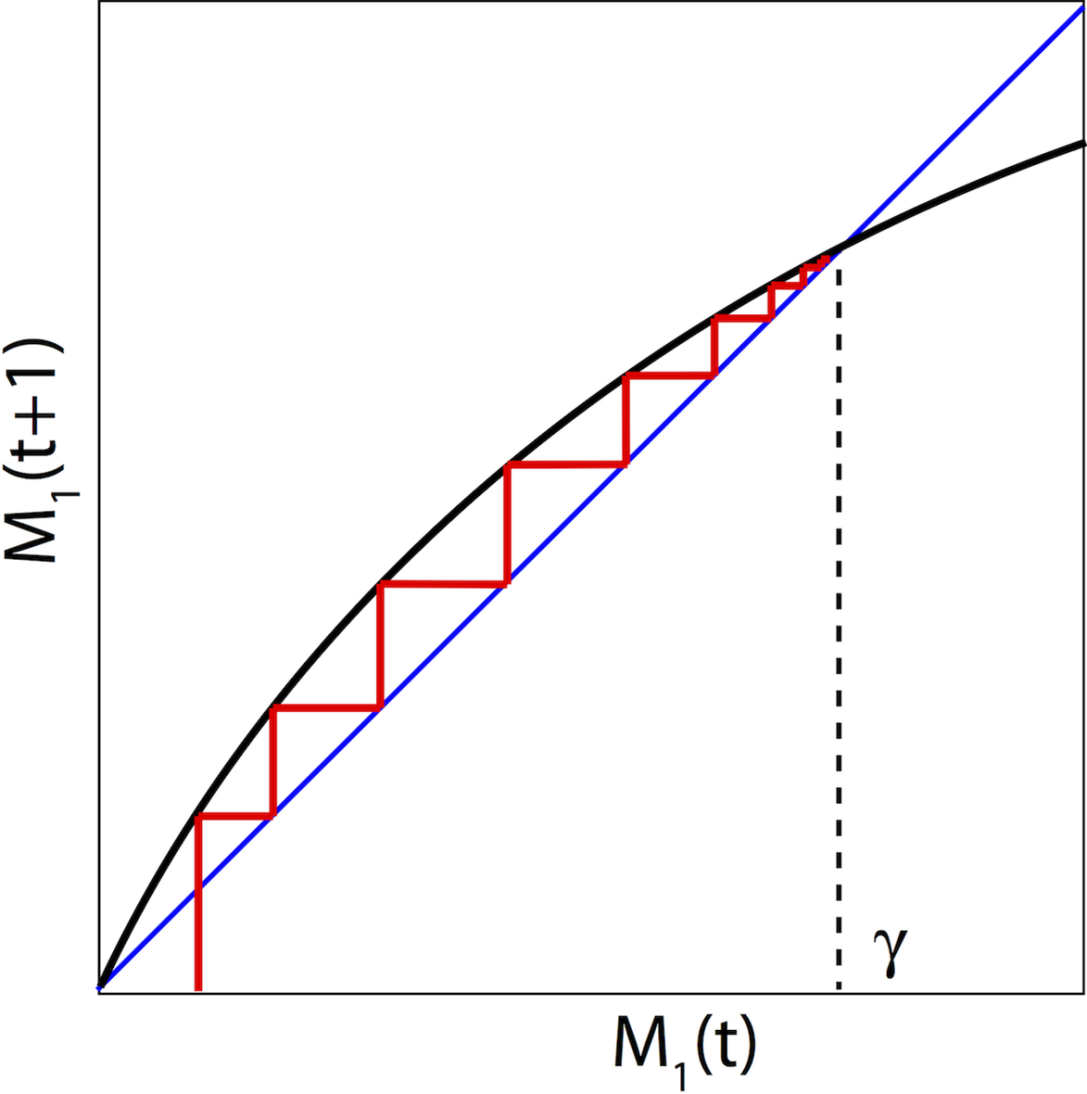
The cobweb diagram of the Beverton-Holt model of monomer's production in the absence of aggregation. The size of monomers increases over time and reaches its carrying capacity γ.

We now take $f(M_{1}(t))={\delta M}_{1}(t)[1-\frac{M_{1}(t+1)}{\gamma }]$. In case of no interaction, the first equation becomes, $M_{1}(t+1)=\frac{{(\delta +1)\gamma M}_{1}(t)}{\gamma +\delta M_{1}(t)}$, which is the popular Beverton-Holt model and is illustrated in Fig 2.

**Fig 3:**
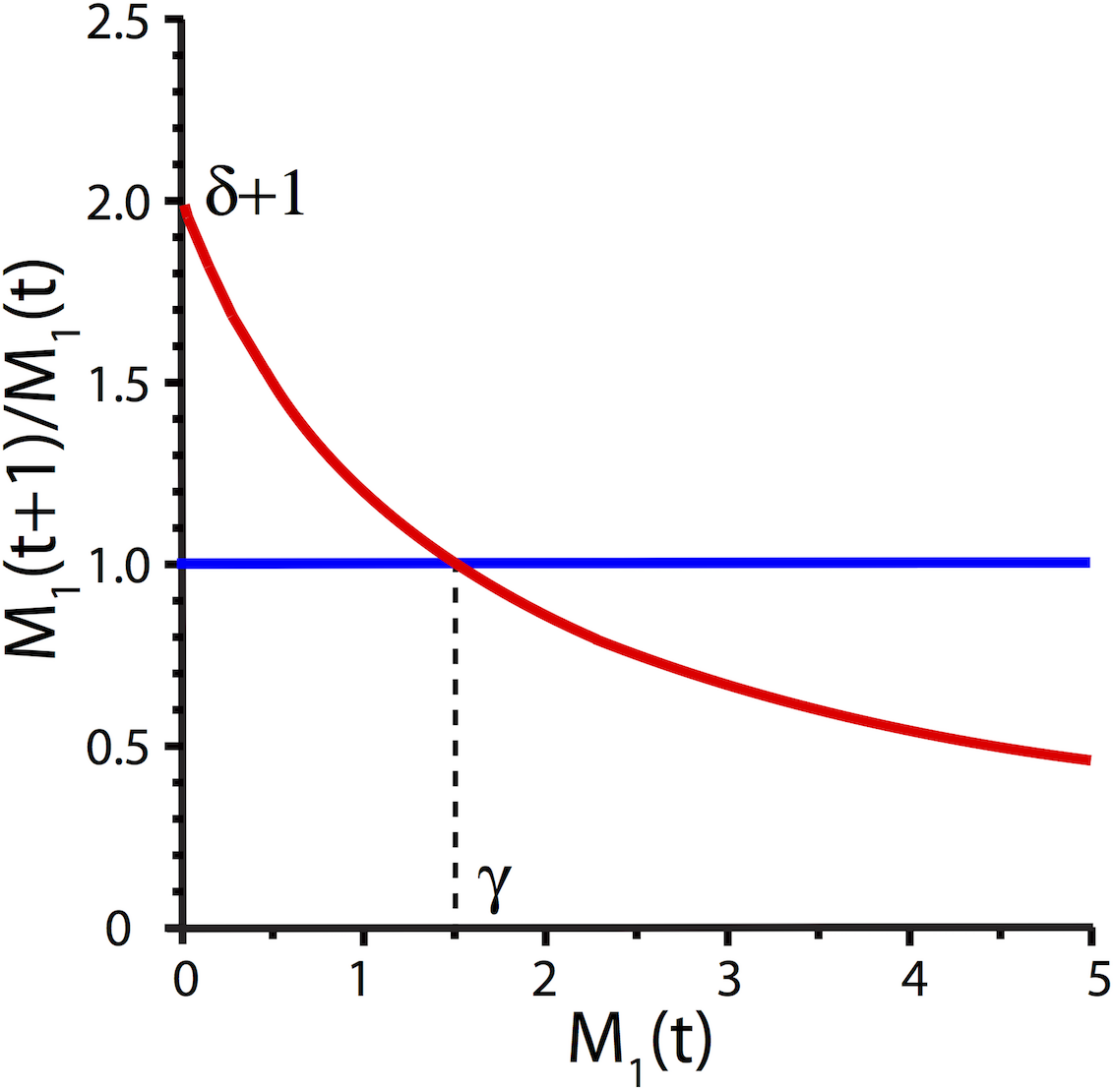
The figure shows the function $M_{2}=\frac{M_{1}(t+1)}{M_{1}(t)}$ (on the vertical axis) versus M_1_(t) (on the horizontal axis). The growth rate decreases from δ + 1 when M_1_ is small, to γ and eventually close to zero.

The Beverton-Holt model [50] is a discrete-time population model which gives the population size (density) *M*_1_(*t* + 1) as a function of the size (density) of the previous generation *M*_1_(*t*). Note that (*δ* + 1) represents the average growth of monomer production with *δ*>0 and *γ* is the carrying capacity. Fig 3 illustrates the growth rate behavior [50, 46].

Using this, we obtain,

$$M_{1}(t+1)=\frac{\left(\delta +1)\gamma M_{1}(t\right)}{\gamma +\delta M_{1}(t)+2\gamma K_{1}M_{1}(t)+\gamma \sum_{t=2}^{n-1}K_{i}M_{i}(t)}$$

$$M_{2}(t+1)=\frac{M_{2}(t)+K_{1}M_{1}^{2}(t)}{1+K_{2}M_{1}(t)}$$

$$M_{3}(t+1)=\frac{M_{3}(t)+K_{2}M_{1}(t)M_{2}(t)}{1+K_{3}M_{1}(t)}$$

⋮

$$M_{n-1}(t+1)=\frac{M_{n-1}(t)+K_{n-2}M_{1}(t)M_{n-2}(t)}{1+K_{n-1}M_{1}(t)}$$ (10)

The basic equilibria are the extinction point E^*^ = (0,0,…,0) and the coexistence point $M^{*}=(M_{1}^{*},\;M_{2}^{*},\;\ldots ,\;M_{n-1}^{*})$, where $M_{1}^{*}=\frac{\delta \gamma }{\delta +n\gamma K_{1}},\;M_{2}^{*}=\frac{K_{1}}{K_{2}}M_{1}^{*},\ldots ,\;M_{n-1}^{*}=\frac{K_{1}}{K_{n-1}}M_{1}^{*}$.

Moreover, all the points on the surface $M_{1}^{*}=0$ are equilibrium points that are of no practical interest since in this case, no chemical reaction is present.

## Stability analysis

As mentioned earlier, [43], it is accepted that 6 monomers are needed to aggregate to make one oligomer. In model (10), let us assume that n = 6. Hence we have the two equilibria E^*^ = (0,0,0,0,0), and $M^{*}=(M_{1}^{*},M_{2}^{*},M_{3}^{*},M_{4}^{*},M_{5}^{*})$. The Jacobian matrix of (10) is now given by

$$J(E^{*})=\left(\begin{array}{ccccc} \left(\delta +1\right) & 0 & 0 & 0 & 0\\ 0 & 1 & 0 & 0 & 0\\ 0 & 0 & 1 & 0 & 0\\ 0 & 0 & 0 & 1 & 0\\ 0 & 0 & 0 & 0 & 1 \end{array}\right)$$

Thus E^*^ is unstable, since *δ* > 0. Next we compute *J*(M^*^).

$$J\left(M^{*}\right)=\left(\begin{array}{ccccc} \left(1-\frac{M_{1}^{*}\left(\delta +2\gamma K_{1}\right)}{\gamma \left(\delta +1\right)}\right) & \frac{-M_{1}^{*}K_{2}}{\left(\delta +1\right)} & \frac{-M_{1}^{*}K_{3}}{\left(\delta +1\right)} & \frac{-M_{1}^{*}K_{4}}{\left(\delta +1\right)} & \frac{-M_{1}^{*}K_{5}}{\left(\delta +1\right)}\\ \frac{K_{1}M_{1}^{*}}{1+K_{2}M_{1}^{*}} & \frac{1}{1+K_{2}M_{1}^{*}} & 0 & 0 & 0\\ 0 & \frac{K_{2}M_{1}^{*}}{1+K_{3}M_{1}^{*}} & \frac{1}{1+K_{3}M_{1}^{*}} & 0 & 0\\ 0 & 0 & \frac{K_{3}M_{1}^{*}}{1+K_{4}M_{1}^{*}} & \frac{1}{1+K_{4}M_{1}^{*}} & 0\\ 0 & 0 & 0 & \frac{K_{4}M_{1}^{*}}{1+K_{5}M_{1}^{*}} & \frac{1}{1+K_{5}M_{1}^{*}} \end{array}\right)$$

To determine the stability of M^*^, we are going to use the following result due to Gerschgorin [51].

### Theorem (Gerschgorin)

Let *A* = (*a*_*ij*_) be a *k* × *k* matrix. Let *S*_*i*_ be the disk in the complex plane with center at *a*_*ij*_, and radius $r_{i}=\sum_{j\neq i}^{k}\left|a_{ij}\right|$. Then all eigenvalues of A lie in $S=\cup_{i=1}^{k}S_{i}$.

To apply this result, let us determine the disks $a_{11}=1-\frac{M_{1}^{*}(\delta +2\gamma K_{1})}{\gamma (\delta +1)}$,

$$r_{1}=|a_{12}|+|a_{13}|+|a_{14}|+|a_{15}|=\frac{(\sum_{i=2}^{n-1}K_{i})M_{1}^{*}}{\gamma (\delta +1)}$$

We need to show that,

$$1-\frac{M_{1}^{*}(\delta +2\gamma K_{1})}{\gamma (\delta +1)}-\;\frac{(\sum_{i=2}^{n-1}K_{i})M_{1}^{*}}{\left(\delta +1\right)}>-1$$

and

$$1-\frac{M_{1}^{*}(\delta +2\gamma K_{1})}{\gamma (\delta +1)}+\;\frac{(\sum_{i=2}^{n-1}K_{i})M_{1}^{*}}{\left(\delta +1\right)}<1$$

Hence

$$M_{1}^{*}\left(\frac{\sum_{i=2}^{5}K_{i}}{\delta +1}+\frac{\left(\delta +2\gamma K_{1}\right)}{\gamma (\delta +1)}\right)<2$$ (11)

and

$$\frac{\sum_{i=2}^{5}K_{i}M_{1}^{*}}{\delta +1}<\frac{M_{1}^{*}(\delta +2\gamma K_{1})}{\gamma (\delta +1)}$$ (12)

substituting condition (11) into (12) and letting $M_{1}^{*}=\frac{\delta \gamma }{\delta +n\gamma K_{1}}$, we obtain,

$$\frac{\delta \gamma }{\left(\delta +1)(\delta +6\gamma K_{1}\right)}\left[\frac{\left(2\delta +4\gamma K_{1}\right)}{\gamma }\right]=2\left[\frac{\delta +2\gamma K_{1}}{\left(\delta +1)(\delta +6\gamma {K_{1}}_{\;}\right)}\right]<1$$ (13)

which is true. Hence, the first condition for the eigenvalue *λ*_1_ to be inside the unit disk is

$$\sum_{i=2}^{5}K_{i}=\frac{\delta +2\gamma K_{1}}{\gamma }$$ (14)

For $2\leq i\leq 5;\;a_{ii}=\frac{1}{1+{K_{i}M}_{1}^{*}}$ and $r_{i}=\frac{K_{i-1}M_{1}^{*}}{1+{K_{i}M}_{1}^{*}}$ we need

$$\frac{1}{1+{K_{i}M}_{1}^{*}}+\frac{K_{i-1}M_{1}^{*}}{1+{K_{i}M}_{1}^{*}}<1$$ (15)

or,

$${1+K}_{i-1}M_{1}^{*}<1+{K_{i}M}_{1}^{*}$$

or,

$$K_{i-1}<K_{i}$$ (16)

and

$$\frac{1}{1+{K_{i}M}_{1}^{*}}-\frac{K_{i-1}M_{1}^{*}}{1+{K_{i}M}_{1}^{*}}>-1$$ (17)

$${1-K}_{i-1}M_{1}^{*}>-1-{K_{i}M}_{1}^{*}$$ (18)

resulting in,

$$M_{1}^{*}(K_{i-1}-K_{i})<2$$ (19)

which is true assuming (16).

Hence the condition that all eigenvalues *λ*_*i*_, 2 ≤ *i* ≤ *n* − 1 lie inside the unit disk is *K*_*i*−1_ ≤ *K*_*i*_, 2 ≤ *i* ≤ *n* − 1

The following theorem summarizes the above stability analysis.

### Theorem

The following statements hold true

(i) E^*^ is always unstable

(ii) M^*^ is stable if

$$\sum_{i=2}^{n-1}K_{i}<\frac{\delta +2\gamma K_{1}}{\gamma }$$ (20)

and *K*_*i*−1_ ≤ *K*_*i*_ for 2 ≤ *i* ≤ *n* − 1

Now to prevent the aggregation of monomers to oligomers and reduce the toxicity level of the neuron cells, one should make the system unstable.

This can be accomplished by either (i) using a catalyst that would reduce *K*_1_ so that

$$\sum_{i=2}^{5}K_{i}>\frac{\delta +2\gamma K_{1}}{\gamma }$$ (21)

and, consequently, the equilibrium point M^*^ is unstable, or (ii) using a suppressant to limit the production of monomers to the effect that the net reproduction rate *δ* is reduced.

Another interesting problem is to determine exactly how many monomers are needed to make an oligomer. As mentioned earlier, we followed the literature and assumed that this number is 6.

However, one may use the formula $M_{1}^{*}=\frac{\delta \gamma }{\delta +n\gamma K_{1}}$ to find the number of monomers needed to make an oligomer, as

$$n=\frac{\delta (\gamma -M_{1}^{*})}{K_{1}M_{1}^{*}}$$ (22)

**Fig 4:**
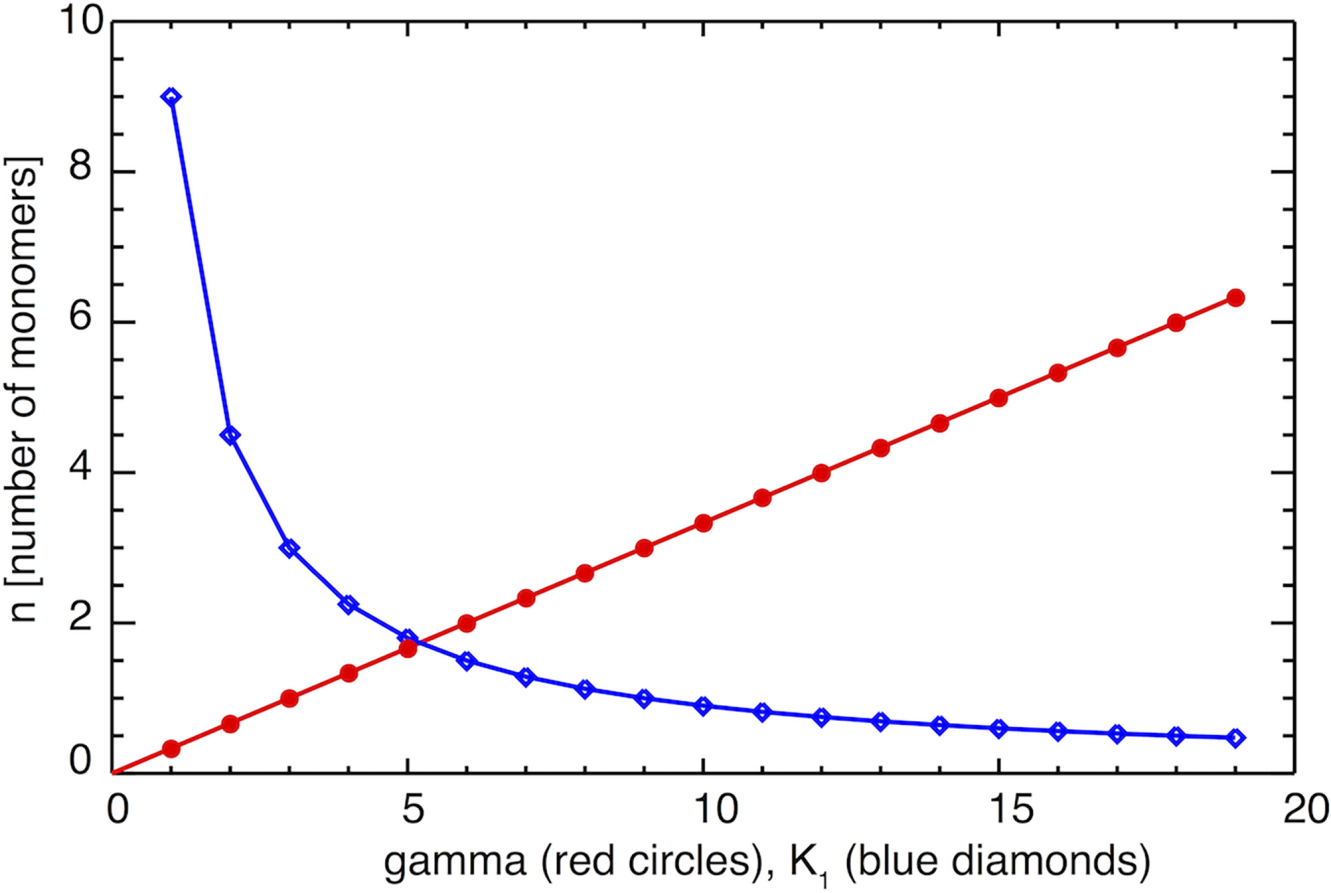
The variation of n, the number of monomer that aggregates to an oligomer, relative to *K*_1_ and *γ* increases or *K*_1_ decreases.

Fig 4 shows the behavior of the number of monomers (n) needed to create an oligomer as a function of *K*_1_ (with gamma fixed; blue curve) and as a function of gamma (with *K*_1_ fixed; red curve).

As can be seen, to increase n, we can either increase *γ* or lower *K*_1_. By increasing n, one may reduce the number of oligomers, and consequently, may decrease the likelihood of developing the Alzheimer's disease.

## Conclusion

AD is an irreversible progressive degenerative disorder that is characterized by the loss of synapses and neurons from the brain and by the presence of extracellular protein containing deposits, with β − Amyloid as the principal protein component. The presence of β − Amyloid is strongly suggested to be a causative of neural degeneration as celebrated by the celebrated Aβ hypothesis. β − Amyloid monomers aggregate to oligomers and in turn oligomers aggregate to fibrils. In this paper, we have developed a discrete mathematical model for the aggregation of monomers to oligomers. The model is based on the assumption that oligomers are the toxic stage of the aggregation, and is built using concepts from chemical kinetics and population dynamics. Based on the model, we propose a mechanism to slow down the aggregation from monomers to oligomers,

$$\sum_{i=2}^{5}K_{i}>\frac{\delta +2\gamma K_{1}}{\gamma }$$

Here, *γ* is the carrying capacity of the β − Amyloid, *δ* is its growth rate, and *K*_*i*_ is the reaction constant of *i* monomers (for instance, *K*_1_ is for a monomer, *K*_2_ is for a diamer,…etc).

Furthermore, we develop an equation for the number of monomers needed to form an oligomer

$$n=\frac{\delta (\gamma -M_{1}^{*})}{K_{1}M_{1}^{*}}$$

where $M_{1}^{*}$ is the equilibrium state of monomers.

In this paper, we present a formula for the reduction of the aggregation of monomers to the toxic oligomers, a process thought to contribute to the development of Alzheimer Disease. To our knowledge, this is the first discrete mathematical modeling study that combines population dynamics and kinetics principles to develop a prevention mechanism that would potentially reduce the risk of Alzheimer Disease. We note that this is a mathematical model that has not been tested or implemented in a clinical trial. We derive formulations for critical parameters thought to affect the progression of the AD. Validating the model is beyond the scope of this paper and is a possible venue for future follow-up work.
